# Emodin Inhibited Pathological Cardiac Hypertrophy in Response to Angiotensin-Induced Hypertension and Altered the Gut Microbiome

**DOI:** 10.3390/biom13091274

**Published:** 2023-08-22

**Authors:** Levi Evans, Tori Price, Nathaniel Hubert, Julia Moore, Yiqui Shen, Maheshi Athukorala, Steven Frese, Kristina Martinez-Guryn, Bradley S. Ferguson

**Affiliations:** 1 Department of Nutrition, University of Nevada, Reno, Reno, NV 89557, USAsfrese@unr.edu (S.F.); 2 Environmental Sciences Program, University of Nevada, Reno, Reno, NV 89557, USA; 3 Biomedical Sciences Program, Midwestern University, Downers Grove, IL 60515, USA; 4 Center of Biomedical Research Excellence for Molecular and Cellular Signal Transduction in the Cardiovascular System, University of Nevada, Reno, Reno, NV 89557, USA

**Keywords:** emodin, microbiome, hypertension

## Abstract

Objective: Evidence suggests that food bioactives affect the epigenome to prevent pathological cardiac hypertrophy. Recently, we showed that emodin, an anthraquinone, attenuated pathological cardiac hypertrophy and histone deacetylase (HDAC) activity. However, we only examined the cardioprotective effects of emodin’s parent compound and not those of emodin metabolites or of emodin–gut microbiome interactions. The microbiome has emerged as a key player in chronic diseases such as metabolic and cardiac disease. Thus, we hypothesized that emodin could reverse hypertension-induced changes in microbial communities. Methods: Normo- and hypertensive (angiotensin II) C57/BL6 female mice were randomly assigned to receive a vehicle (Veh; DMSO:PEG 1:1) or emodin (Emod; 30 mg/kg) for 14 days. Body weights were collected pre- and post-treatment, and blood pressure was assessed via tail cuff. At the study’s end, the mice were euthanized and assessed for their heart weights. In addition, stool samples and cecal contents were collected to elucidate changes in the microbial populations using 16S rRNA sequencing. Lastly, the tissue was lysed, and RNA was isolated for qPCR. One-way ANOVA with Tukey’s post hoc test was performed unless otherwise specified, and *p* < 0.05 was considered significant. Results: Emodin significantly attenuated cardiac hypertrophy in the female mice. No significant changes were observed in body weight or systolic blood pressure in response to hypertension or emodin. Lastly, analysis suggests that hypertension altered the microbiome in the cecum and cecal content, with additional evidence to support that emodin affects gut microbiota in the feces and colon. Conclusions: Our data demonstrate that emodin attenuates pathological hypertrophy in female mice. Future research is needed to dissect if changes in the microbiome contributes to emodin-mediated attenuation in cardiac remodeling.

## 1. Introduction

Hypertension is a major public health concern that contributes to the development of heart failure [1]. Hypertension impacts vascular resistance and loads the heart, which contributes to scar tissue formation (fibrosis) and heart enlargement (hypertrophy). This ultimately results in cardiac dysfunction and heart failure [1]. Technological advancements have enhanced our understanding of diet in (cardiovascular disease) CVD and health. This is evident in recent studies of the microbiome, which has emerged as a major player in CVD pathogenesis [2,3,4]. In fact, distinct microbial communities can be found in humans at risk of CVD [5] with dyslipidemia [2], atrial fibrillation [6], hypertension [4], and heart failure [7]. More importantly, angiotensin II-induced hypertension only increased pathological cardiac hypertrophy, fibrosis, and dysfunction in conventionally raised mice but not germ-free mice, suggesting that the microbiome is essential for hypertension-induced changes in the myocardium [8]. Consistent with this, fecal microbiota transplant (FMT) from hypertensive rats led to hypertension and dysbiosis in non-hypertensive rats [9]. Conversely, therapies that target and normalize the microbiome, similar to healthy controls, improve hypertension in rodents and humans [10,11,12]. As the diet plays a major role in regulating the microbiome, dietary components have the potential to regulate and maintain microbial homeostasis to prevent or ameliorate CVD and myocardial dysfunction.

According to the Global Burden of Disease study, poor-quality diets have contributed to the rise in hypertension, heart failure, and other cardiovascular disease components [13]. Indeed, diet is now considered the number one risk factor for preventable diseases in the United States and other developed nations [13]. Understanding how nutrients and other dietary components regulate cellular fate to promote health and longevity affords clinicians an opportunity to identify dietary strategies that can reduce and potentially reverse CVDs including hypertension and heart failure. This can be seen in the literature where the increased intake of fruits and vegetables is linked to cardioprotection [14,15], and in which studies identified fiber, vitamins, minerals, and phytochemicals within these foods as capable of preventing and potentially treating pathological cardiac hypertrophy, fibrosis, and dysfunction [16,17,18]. 

Phytochemicals have emerged over the last decade for the prevention and treatment of CVD [19,20]. Indeed, our group has published that emodin, found in rhubarb, buckthorn, knotweed, beans, and cabbage, can inhibit pathological cardiac remodeling [21]. Other groups have also highlighted the cardioprotective actions of emodin in the heart. For instance, Wu et al. [22] reported that emodin improved left ventricular function via increased phosphorylation of protein kinase B/glycogen synthase kinase-3 beta (pAkt/pGSK-3β) in diabetic rats with cardiomyopathy [22]. Emodin was also shown to attenuate activation of the NLR family pyrin domain containing 3 (NLRP3) inflammasome that led to decreased cardiomyocyte death in response to ischemia-reperfusion (I/R) injury [23]. As mentioned above, we reported emodin inhibited pathological cardiomyocyte hypertrophy by attenuating Zn-dependent HDACs, while others have shown improvements via activation of NAD^+^-dependent HDACs [21,24], thus indicating that emodin can regulate the genome via epigenetic interactions.

Historically, many studies focused on the actions of the parent compound, e.g. emodin, and examined direct actions within cell culture or through intraperitoneal injections. Few studies have examined the impact of emodin on the microbiome [25], nor have these studies elucidated emodin-microbiome interactions in a model of CVD. This is an important consideration given the recent evidence highlighting the role for phytochemicals in the regulation of the gut microbiome [26]. Indeed, several recent reports have highlighted the potential for phytochemical-gut microbiome interactions that change microbial diversity, and attenuate gut inflammation and dysbiosis, which can impact cardiovascular phenotypes that include improving arterial stiffness commonly linked with aging, obesity, and hypertension [26]. 

As the microbiome has been implicated in modulating phytochemical efficacy [27], it is imperative to elucidate the effects of emodin in the gut. Thus, the goal of this study was to examine the effects of emodin on the microbiome of hypertensive and healthy mice. Again, we observed that emodin attenuated pathological hypertrophy in hypertensive female mice with no changes in blood pressure. We also report several novel and interesting observations in this study. First, hypertension decreased microbial diversity and altered the microbiome, in which the abundance of bacteria like *Lachnospiraceae x4554* was negatively correlated with heart size. Second, emodin decreased bacterial diversity in healthy mice three days post-ingestion yet increased the abundance of beneficial bacteria (e.g., *Bacteroides thetaiotamicron*). Third, emodin normalized the microbiome in hypertensive animals similar to the control mice and improved bacterial composition, which favored leanness (e.g., *Roseburia* or *Akkermansia*) and cardioprotection (e.g., *Akkermansia*). Lastly, the microbial composition was dependent on the route of emodin administration, in which the gavage-dosed animals displayed an increase in selective protective bacteria (e.g., *Roseburia*), while the intraperitoneally dosed mice exhibited an increase in other protective bacterial species (e.g., *Bacteroides thetaiotamicron*), suggesting that liver and bacterial metabolism of emodin affect microbial communities. While correlative, these findings suggest that the cardioprotective actions of emodin are driven, in part, through the regulation of the microbiome.

## 2. Materials and Methods

Animal experiments: All animal studies were performed in accordance with the University of Nevada Reno IACUC. Female C57BL/6 mice were purchased from Jackson Labs and arrived at nine weeks of age. The animals were housed in the Office of Animal Resources at the University of Nevada in Reno. One week post acclimation, the mice (10 weeks of age) were randomly assigned to receive a sham operation (Sham) or a subcutaneous 14-day micro-osmotic pump (Alzet, Cupertino, CA, model 1002) that contained angiotensin II (AngII, Bachem, Bubendorf, Switzerland), which was administered at 1.5 μg/kg/min. The mice that were assigned to receive AngII were further randomly assigned to receive an intraperitoneal (i.p.) injection of a vehicle control (1:1 dimethyl sulfoxide:polyethylene glycol-300 (DMSO, Pharmco-AAPER; PEG-300, Acros Organics, Waltham, MA) or emodin (SelleckChem, Houston, TX, S2295) at 30 mg/kg/day (dissolved in a 1:1 solution of DMSO:PEG-300)). The Sham group further received a vehicle control via IP injection. The 14-day experiment contained three different groups: non-hypertensive sham control (Sham); hypertension plus vehicle control (Hypert); and hypertension plus emodin at 30 mg/kg/day (Hypert + Emodin). In all, n = 8 mice/group were used in these studies. The mice were weighed and dosed with a vehicle or emodin via IP injection each day for 14 days. The mice were acclimated to a tail-cuff blood pressure system (Coda High Throughput System, Kent Scientific, Torrington, CT) for 3 days prior to systolic blood pressure measurements at the study’s end. The ceca and hearts were dissected at the end of the study. The whole heart and left ventricle weights were assessed. The tibia was dissected and measured. The heart weight (HW) and left ventricle (LV) weight were normalized to the tibia length as well as the body weight. The ceca and ceca content were used for 16S rRNA sequencing (n = 3/group).

Nine-week-old C57BL/6 mice were ordered from Jackson Labs and allowed to acclimate. Ten-week old healthy mice were randomly assigned to receive the vehicle (1:1, DMSO:PEG-300) or emodin at 30 mg/kg/day via oral gavage for seven days. The mice were weighed and dosed every day for seven days. Fecal samples were collected every day for seven days. In total, n = 4/treatment group were used for the gavage studies. At the study’s end, the gastrointestinal tract was dissected into individual components of the small and large intestine as well as the cecum. Tissues were immediately flash-frozen and sent to Midwestern University for DNA extraction and 16S sequencing. Daily feces were also analyzed via 16S rRNA amplicon sequencing.

Real-time quantitative polymerase chain reaction (rt-qPCR): RNA was isolated from the left ventricles of the mice from the hypertension experiments via QIAzol (Qiagen, Hilden, Germany) as previously described [21]. The RNA concentration was determined via a NanoDrop Spectrometry ND1000, and 500 ng RNA was reverse transcribed to cDNA using a Verso cDNA Synthesis Kit (ThermoFisher Scientific, Waltham, MA). The RT-qPCR was examined with Apex qPCR GREEN Master Mix (Genesee Scientific, 42–120) using primers for atrial natriuretic peptide (ANP) (GCC GGT AGA AGA TGA GGT CAT, GCT TCC TCA GTC TGC TCA CTC) and B-type natriuretic peptide (BNP) (CGC TGG GAG GTC ACT CCT AT, GCT CTG GAG ACT GGC TAG GAC TT). Gene expression was examined using the BioRad CF96X real-time instrument.

DNA extraction and 16S rRNA sequencing: The small intestine was separated into the duodenum (first 4 cm distal of the stomach), jejunum (beginning 6 cm distal of the stomach, with the next 6 cm collected as well), and ileum (8 cm proximal of the cecum, with the next 6 cm collected as well). These tissues as well as the colon and cecum were placed in o-ring tubes with silica beads along with a DNA lysis buffer and bead beaten for 2 min. Subsequently, DNA extraction was performed as previously reported [28]. The DNA was submitted to Argonne National Laboratory for 16S rRNA amplicon sequencing conducted via Illumina Mi-Seq. Quality filtering and downstream analyses were performed using previously published protocols [29]. Any sequences that were found to be unknown were blasted to determine their origins. If sequences were determined to be eukaryotic, then they were removed from the analysis. Illumina-utils software was used to demultiplex, merge paired reads, and quality filter sequence data. Minimum entropy decomposition was used to assess the quality-filtered reads. High-resolution oligotypes were generated using Shannon entropy [30]. GAST was used to assign taxonomies to oligotypes as previously described [29]. Shannon diversity analysis was performed using QIIME software. Anvi’o was used to generate heat maps displaying taxonomic differences between the diet and treatment groups. The normalized relative taxonomic abundance at the family level was compared via Kruskal–Wallis testing based on (1) diet and (2) treatment, encompassing all gut regions and also within each gut region (FDR correction *p* < 0.05) in QIIME software. In some cases, groups contained n = 2 due to samples failing the sequencing run or having too few sequences to be incorporated into the analysis. Uploaded data: https://www.ncbi.nlm.nih.gov/bioproject/PRJNA1003954 (accessed on 9 August 2023).

Statistical analysis: Statistical differences in gene expression and gross anatomy was determined via one-way ANOVA with Tukey’s post-hoc analysis to compare between groups. Significance was set at *p* < 0.05. Statistical analyses for 16S sequencing are described in the previous section. 

## 3. Results

### 3.1. Emodin Inhibits Pathological Cardiac Hypertrophy in Response to Hypertension

Angiotensin II (AngII) participates in the renin angiotensin system (RAS) and targets angiotensin receptors, which subsequently activates vasoconstriction and the signal transduction pathways in the heart [31]. As a result, AngII induces systemic hypertension and causes hallmarks of heart failure, including cardiac hypertrophy and fibrosis [31]. To begin our studies, we tested the postulate that emodin would attenuate hypertension-induced pathological cardiac hypertrophy. Our data demonstrate that the mice treated with AngII experienced an increase in systolic blood pressure, heart weight (HW), and left ventricular weight (LVW) compared with the sham vehicle control group (Sham) (Table 1). Moreover, the HW and LVW remained significantly increased when normalized to either the tibia length or body weight (BW) (Table 1). Consistent with this increase in cardiac hypertrophy, we further demonstrate that the hypertensive mice had increased gene expression of hypertrophy markers, natriuretic peptides ANP and BNP (Table 1). Notably, i.p. delivery of emodin (Hypert + Emodin) attenuated the HW and LVW with and without normalization to the tibia length and BW when compared with the hypertensive mice (Table 1). No changes were observed in BW or systolic blood pressure between the emodin-treated and hypertensive mice (Table 1). Emodin further attenuated the upregulation of fibrotic genes collagen 1 (Col1) and connective tissue growth factor (CTGF) (Table 1). These data suggest that emodin was cardioprotective in this hypertension model.

### 3.2. Hypertension and Pathological Cardiac Hypertrophy Are Linked to Changes in the Gut Microbiome and Normalized with Emodin

**Figure 1:**
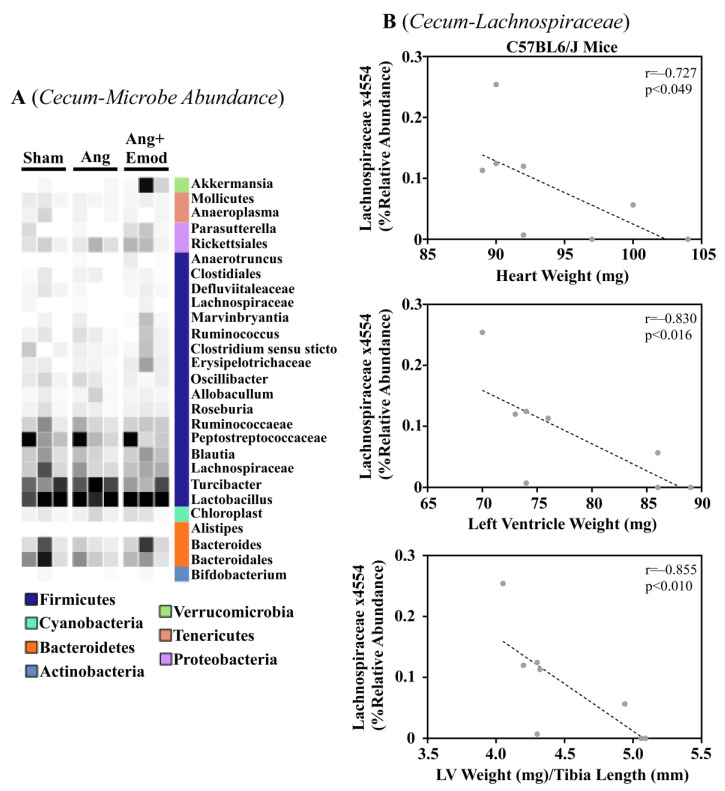
Angiotensin II-induced changes in the cecum microbiome were altered with emodin treatment. Ten-week-old C57BL6/J male mice were treated with vehicle (DMSO:PEG 200), angiotensin II (Ang) (1.5 g/kg/min), or Ang + emodin (Ang + Emod; 30 mpk). Cecal content was removed and cecum collected for 16S rRNA sequencing. (**A**) A heatmap showing changes in the microbiome and (**B**) Spearman correlation analysis performed to identify bacteria that negatively correlated with heart weight, left ventricle (LV) weight, and LV weight normalized to tibia length (LV/TL). Color intensity indicates abundance in the heatmap, with darker colors representing more abundance.

**Figure 2:**
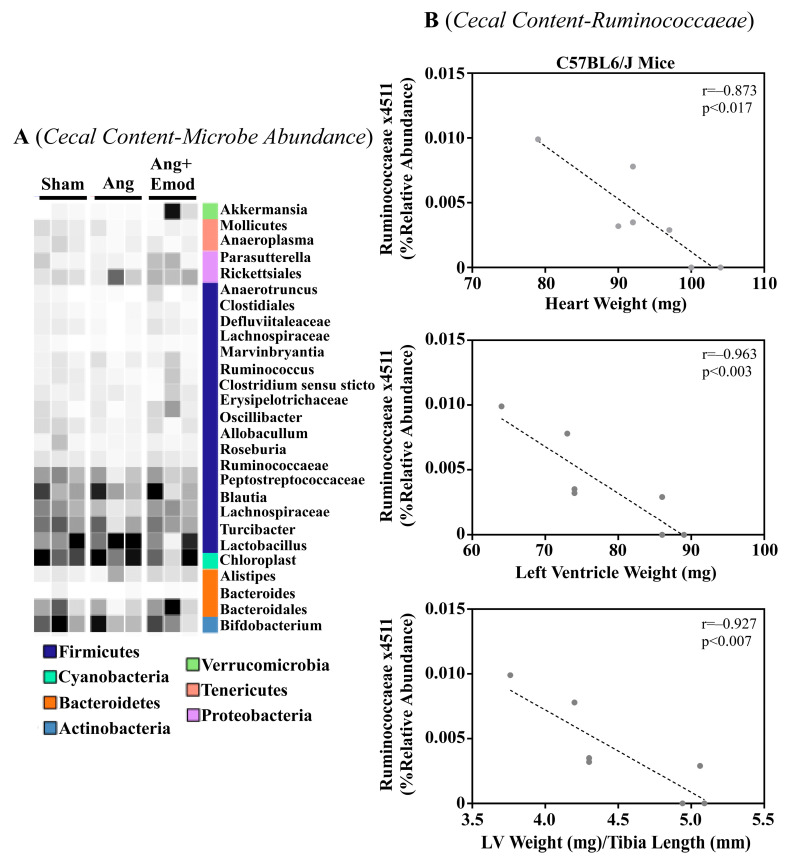
Angiotensin II-induced changes in the cecal microbiome were altered with emodin treatment. Ten-week old C57BL6/J male mice were treated with vehicle (DMSO:PEG 200), Angiotensin ii (Ang) (1.5 g/kg/min) or Ang + emodin (Ang + Emod; 30 mpk). Cecal content was scraped from the cecum and cecal content sent for 16S rRNA seqeuncing. (**A**) A heatmap showing changes in the microbiome and (**B**) Spearman correlation analysis was performed to identify bacteria that negatively correlated with heart weight, left ventricle (LV) weight and LV weight normalized to tibia length (LV/TL). Color intensity indicates abundance in the heatmap, with darker colors representing more abundance.

The microbiome has been implicated in CVD development [8]. Moreover, it has been suggested that phytochemicals interact with the microbiome to protect the heart [27]. As we observed that emodin attenuated hypertension-induced cardiac hypertrophy, we next sought to examine how emodin regulated microbes within the gut of hypertensive mice. Using 16S sequencing, we report altered microbial composition in the ceca of hypertensive animals and hypertensive mice treated with emodin (Figure 1A). Moreover, we report that *Lachnospiraceae x4554* was negatively correlated with the heart weight, left ventricle (LV) weight, and LV weight normalized to the tibia length (Figure 1B). Interestingly, emodin normalized *Lachnospiraceae x4554* back to levels similar to the healthy (Sham) mice (Figure 1A). We further observed that *Akkermansia*, which has been previously associated with anti-obesity and anti-diabetes properties [32,33], was significantly increased in the emodin-treated hypertensive mice compared with the hypertensive mice in the cecum (Figure 1A) and cecal content (Figure 2A). Of note, we further observed that hypertension and emodin altered the microbial abundance of *Ruminococcaeae* in the cecal content and that *Ruminococcaeae* was negatively correlated with pathological cardiac hypertrophy (Figure 2B). Combined, these data suggest that hypertension or changes in heart size may negatively affect the microbiome and that emodin can normalize the expression of protective bacteria (e.g., *Akkermansia*).

### 3.3. Emodin Rapidly Changes Microbial Diversity and Bacterial Composition throughoutthe Gut

**Figure 3:**
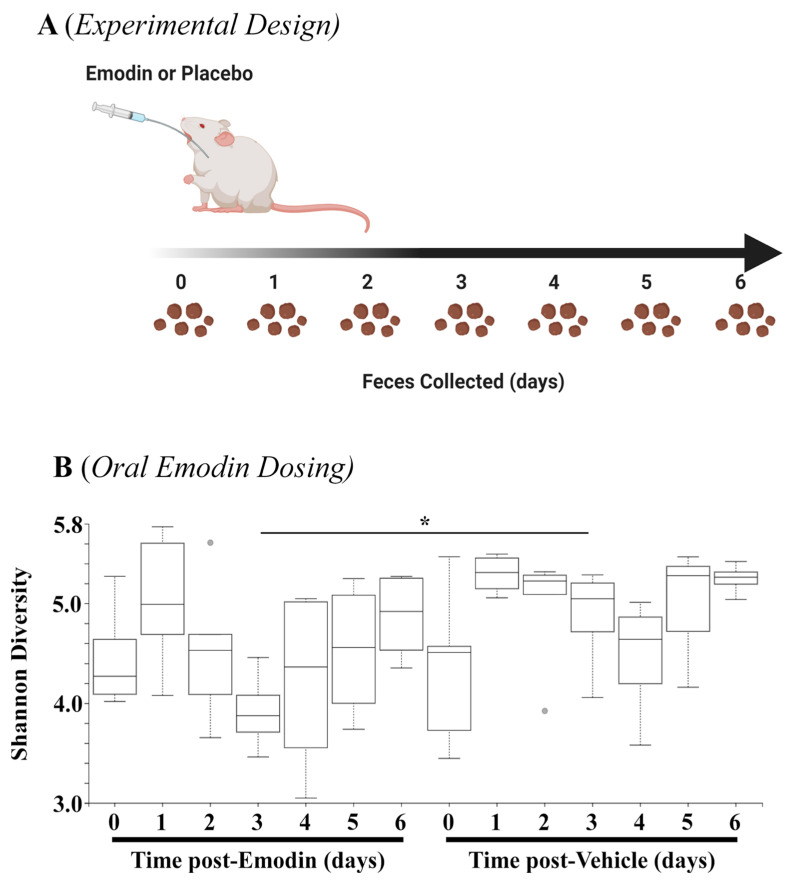
Fecal microbial diversity was attenuated with emodin. (**A**) Ten-week-old C57BL6/J mice were gavaged with vehicle control (DMSO:PEG) or emodin (30 mpk) daily with feces collected daily. (**B**) Shannon diversity analysis was performed using QIIME software for days 0–6 of the experiment comparing emodin (n = 4) vs. vehicle control (n = 5) (day 3, H = 4.9, * *p* = 0.0275).

The data above demonstrate that hypertension affects the microbiome, and emodin can normalize these pathogenic actions. As a next step, we examined how quickly emodin could alter the microbiome in healthy mice by collecting fecal samples daily from mice gavaged with emodin (30 mg/kg/day) or placebo control for up to seven days (Figure 3A). The 16S sequencing showed that emodin significantly reduced microbial diversity through three days of emodin treatment (Figure 3B). Interestingly, these changes appeared to diminish by day 7, although they were still reduced (Figure 3B). This is in keeping with other findings that changes in the microbiome are dynamic and can return to homeostasis of the host [34,35].

**Figure 4:**
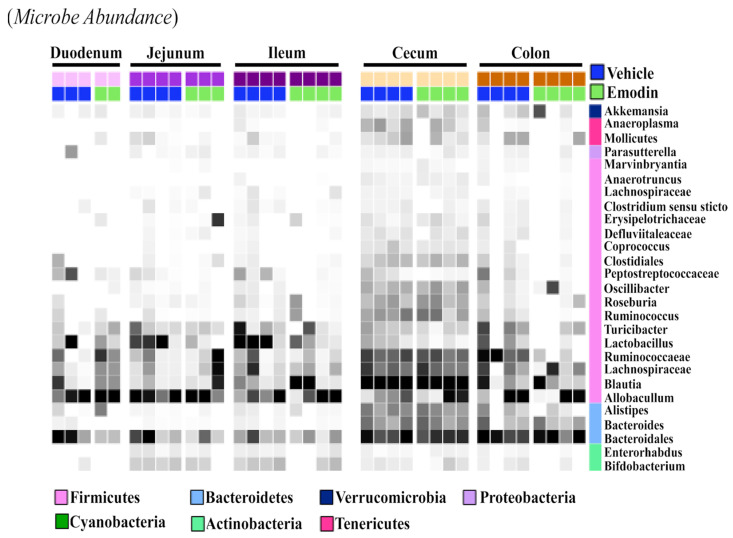
Emodin affected the microbiome of the ceca and colons in mice. 10-week old C57BL6/J mice were gavaged with vehicle control (DMSO:PEG) or emodin (30 mpk) daily and fecal content collected daily. At the study end (day 7) the duodenum, jejunum, ileum, cecum and colon were collected and 16S rRNA sequencing used to examine microbial changes. A heatmap was used to visualize changes across the gut and in response to emodin treatment. Color intensity indicates abundance in the heatmap, with darker colors representing more abundance.

While the fecal samples (Figure 3B), ceca (Figure 1), and cecal content (Figure 2) suggest that emodin alters the microbiome, it does not show if these changes are localized or take place throughout the digestive tract. As such, we investigated the effects of emodin on microbial communities throughout the entire GI tract. We report that emodin reduced the abundances of select taxa in the duodenum, jejunum, ileum, cecum, cecum content, colon, and feces after seven days of dosing (Figure 4). For example, *Lactobacillus* was enriched throughout the entire GI tract and in the feces of the vehicle-treated mice but not in the emodin-treated mice (Figure 4). Furthermore, emodin reduced *Peptostreptococcaceae* in the duodenum, ileum, jejunum, and in the colon (Figure 4). Notably, an increased abundance of *Peptostreptococcaceae* has been observed in diet-induced obese mice in the jejunum [29]. Not all of these changes were generalizable across the gut where, for instance, *Ruminococcaceae* was enriched in the colons of the vehicle-treated mice but not the emodin-treated mice (Figure 4). These data demonstrate that emodin can rapidly change the microbiome and that these anti-microbial actions can occur across the GI tract as well as within selective regions of the gut. While emodin decreased the abundance of some microbes, enrichment was also observed. For example, *Allobaculum* was enriched in the jejunum and feces through day 3 for the mice supplemented with emodin. Most notably, emodin increased the taxonomical abundance of *Akkermansia* in the colon and feces (Figure 3B and Figure 4). This also occurred in the ceca of the hypertensive emodin-treated mice above (Figure 1). Additionally, *Roseburia* was enriched by emodin in the jejunum, ileum, and colon (Figure 3). Similar to *Akkermansia, Roseburia* has also been implicated in obesity prevention and heart health, and this has been attributed to increasing short-chain fatty acid production [36].

## 4. Discussion

In this report, we showed that emodin attenuated hypertension-induced cardiac hypertrophy without changing systolic blood pressure or body weight in female mice. This is consistent with a reduction in ANP and BNP, which are two biomarkers of heart failure [37]. In addition, we report novel findings that (1) emodin increased the abundance of cardioprotective bacteria (e.g., *Akkermansia*) in hypertensive mice, and (2) emodin rapidly (within 3 days) decreased microbial diversity while increasing the abundance of cardioprotective bacteria, specifically in the colon (e.g., *Akkermansia*), as well as health-promoting bacteria (e.g., *Roseburia*) throughout the gut. These data show that microbial changes occur rapidly with dietary intervention and that emodin can improve the bacterial abundance of known cardioprotective species in both healthy and hypertensive mice.

Cross-sectional human studies show a relationship between hypertension and the gut microbiome in humans [38,39,40]. Indeed, patients with higher blood pressure or hypertension have a higher abundance of gram-negative bacteria such as Klebsiella, Parabacteroides, and Prevotella [38,39,40]. Moreover, Klebsiella and Streptococcaceae were observed to be positively correlated in patients with high blood pressure [40]. Consistent with this, germ-free (GF) mice dosed with fecal matter from hypertensive donors developed high systolic and diastolic blood pressure compared to GF counterparts with normotensive FMT [3,41]. Lastly, dysbiosis has been described in several hypertensive rodent models, including the angiotensin ii-infused model similar to what we employed in our study design [9,10,42]. Given this, it isn’t surprising that we observed changes in gut bacteria in hypertensive mice, which were either reversed or partially restored with emodin treatment.

*Akkermansia* is a commensal microorganism that has gained much attention; this microbe is negatively correlated with obesity and type 2 diabetes [43]. In fact, probiotic supplementation of *Akkermansia* attenuated obesity and diabetes in rodents and humans [32,33]. As obesity and diabetes both increase the risk of developing CVDs [1], *Akkermansia* is likely to protect the heart by ameliorating metabolic disease. Additionally, *Akkermansia* also acts as an anti-inflammatory microbe; inflammation is a common driver of CVD and heart dysfunction [44,45]. Consistent with this, *Akkermansia* supplementation reduced inflammation-induced damage and atherosclerosis in the hearts of Apolipoprotein E deficient mice fed a Western Diet [46]. It should also be noted that rhubarb, a plant rich in emodin, also increased *Akkermansia* abundance and this was linked to inhibition of metabolic disease [47]. Here, we report that emodin enriched *Akkermansia*, independent of the route of administration (i.e., gavage vs. i.p.), and this increase was linked to attenuation of hypertension-induced cardiac hypertrophy. Combined, our data suggest that emodin acts as a prebiotic of *Akkermansia*. Future studies that look at the cardioprotective actions of *Akkermansia* as a probiotic in our hypertension model would be of interest, as would in vitro analysis to determine if emodin directly improves *Akkermansia* growth.

Similar to *Akkermansia*, *Roseburia* is a microbe that has been linked to health promotion, and a diet rich in complex carbohydrates drives the increase in *Roseburia* abundance [36]. Of interest, *Roseburia* is a short chain fatty acid producing bacteria located in the intestine [48]. Short-chain fatty acids like butyrate have been reported to inhibit histone deacetylase (HDAC) enzymes; butyrate also attenuates pathological cardiac remodeling and improves cardiac function via HDAC inhibition [49,50]. Specifically, orally supplemented sodium butyrate attenuated high-fat diet-induced dysmetabolic symptoms including cardiac dysfunction, hypertrophy, fibrosis and apoptosis in mice [50]. Similar results were reported with streptozotocin-induced diabetic cardiomyopathy [49]. Both studies showed that butyrate inhibited HDAC activity in the heart [49,50]. *Roseburia* supplementation was also shown to decrease atherosclerotic lesions in germ-free mice, improve gut permeability and reduce inflammation compared to control counterparts [51]. Just as interesting, *Roseburia* inoculation increased histone H3 acetylation and gene expression in the gut [51]. However, the authors reported no change in epigenetic marks in the aortas [51]. These data suggest that the butyrate-producing microbe *Roseburia* was able to regulate local but not systemic epigenetic marks. Consistent with these HDAC inhibitory actions, our previous work showed that emodin reduced HDAC activity in the hearts of mice [21]. Moreover, in this report we showed that emodin increased *Roseburia* abundance, suggesting that emodin-mediated HDAC inhibition may, in part, be due to *Roseburia*-induced short chain fatty acids in addition to emodin inhibitory actions. It would be interesting to assess HDAC activity and pathological cardiac remodeling in germ-free mice hypertensive mice treated with emodin and with and without *Roseburia* inoculation.

Unlike the other bacteria discussed above, *Peptostreptococcaceae* was more abundant in mice treated with vehicle control than those gavaged with emodin. The role for *Peptostreptococcaceae* in human health remains unclear. For example, individuals with the autoimmune condition, primary immune thrombocytopenia, had lower levels of *Peptostreptococcaceae* compared to healthy controls [52]. Further, *Peptostreptococcaceae* correlated with circulating N-acyl-ethanolamines and 2-monoacyl-glycerols after omega fatty acid consumption [53]. These two reports would make it seem as if *Peptostreptococcaceae* may confer beneficial outcomes. However, others have shown that *Peptostreptococcaceae* is enriched with high fat diet [54,55], specifically in the jejunum [29]. In fact, *Peptostreptococcaceae* was reduced in ApoE knockout mice that consumed a high fat diet treated with the cholesterol-lowering agent, α-Cyclodextrin [55], suggesting that lowering *Peptostreptococcaceae* is beneficial in this model. Here, we report that emodin reduced *Peptostreptococcaceae* abundance and is cardioprotective, consistent with findings from high fat diet studies. These mixed reports demonstrate that further studies on *Peptostreptococcaceae* in heart health are needed.

Our data also show a strong negative correlation between cardiac remodeling and the relative abundance of *Lachnospiraceae x4554*. The Lachnospiraceae family of microbes, which includes the *Roseburia* and *Ruminococcaceae* genera, is anaerobic and belongs to the Fermicutes phylum [56]. The role of *Lachnospiraceae* in health and disease, including metabolic dysfunction and diseases of the liver, kidney, and gut, was recently reviewed [56]. Discussed therein were several reports that found Lachnospiraceae genera, particularly *Blautia*, were associated with the aforementioned diseases [57,58,59,60]. For example, *Blautia* was enriched in rats with chronic kidney disease induced via a 5/6 nephrectomy [57] which, similar to the angiotensin II model we used, increased cardiac remodeling through hypertension. Indeed, Feng et al. associated the Lachnospiraceae genus *Blautia* with plasma metabolites of disease, including those of cardiovascular disease (e.g., trimethylamine N-oxide (TMAO)), and nephritis [57]. However, at least 58 genera and unclassified strains make up the Lachnospiraceae family of microbes [56], including the cardioprotective bug discussed above, *Roseburia*, and a bug we found to negatively correlate with pathological heart enlargement: *Lachnospiraceae x4554*. Finally, the Lachnospiraceae family of microbes contributes to the production of short-chain fatty acids such as the above characterized HDAC inhibitor butyrate [56]. Therefore, these data and ours suggest that Lachnospiraceae can be both protective and deleterious in the heart, depending on the strain.

## 5. Conclusions

In conclusion, emodin conferred cardioprotection in a model of hypertension and these cardioprotective benefits were positively linked with Akkermansia, Rosuburia, and Allobaculum abundance; and negatively associated with Peptostreptococcaceae enrichment. Moreover, emodin reduced microbiota diversity, which is consistent with reports of other phytochemicals that have been shown to have anti-microbial properties [61]. Lastly, consistent with previous reports, we show that hypertension markedly changed the microbiome, which was partially restored with emodin treatment. While our data suggest that the microbiome plays a role in the anti-hypertrophic actions of emodin, this report is correlative and further research involving work with germ-free animals, FMT and in vitro assays are needed to define the bacteria important for emodin-mediated actions in the heart.

## Figures and Tables

**Table 1:** Anthropomorphic analysis of hypertensive (hypert) mice with and without emodin. One-way ANOVA with Tukey’s post hoc test was used to detect significance. ^a^ *p* < 0.05.

	Hypertension Experiment		
	Sham	Hypert	Hypert ± Emodin
**Study End BW (g)**	21.16 ± 0.87	21.25 ± 0.56	20.09 ± 1.45
**Change in BW (g)**	2.09 ± 0.14	2.43 ± 0.33	1.19 ± 0.25
**Systolic Blood Pressure (mmHg)**	123.98 ± 9.36	149.32 ± 14.77 ^a^	137.59 ± 8.99 ^a^
**Heart Weight (mg)**	83.00 ± 1.82	104.25 ± 3.20 ^a^	79.40 ± 4.39
**Left Ventricle Weight (mg)**	68.00 ± 2.32	86.50 ± 2.33 ^a^	67.00 ± 3.69
**HW (mg)/TL (mm)**	4.76 ± 0.06	6.06 ± 0.14 ^a^	4.55 ± 0.23
**LVW (mg)/TL (mm)**	3.90 ± 0.13	5.03 ± 0.10 ^a^	3.84 ± 0.20
**HW (mg)/BW (g)**	3.92	4.91 ^a^	3.95
**LVW (mg)/BW (g)**	3.21	4.1 ^a^	3.33
**ANP/18S 2^−∆∆Ct^**	1.06 ± 0.25	4.80 ± 2.29 ^a^	2.63 ± 1.82
**BNP/18S 2^−∆∆Ct^**	1.06 ± 0.30	4.11 ± 1.05 ^a^	2.59 ± 1.16
**Col1/18S 2^−∆∆Ct^**	1.00 ± 0.08	1.60 ± 0.18 ^a^	1.11 ± 0.26
**CTFG/18S 2^−∆∆Ct^**	1.03 ± 0.21	3.87 ± 2.06 ^a^	2.12 ± 0.73
**18S 2^−∆∆Ct^**	1.03 ± 0.22	0.84 ± 0.17	0.85 ± 0.16

## Data Availability

Data will be made freely available. See link in methods for Microbiome data.

## Abbreviations

HDAC (histone deacetylase); Veh (Vehicle); Emod (Emodin); CVD (Cardiovascular Disease); Akt/GSK-3β (Protein kinase B/glycogen synthase kinase 3 beta); NLRP3 (NLR family pyrin domain containing 3); I/R (Ischemia/Reperfusion); AngII (angiotensin ii); HW (heart weight); LV (left ventricle); BW (body weight); LVW (left ventricular weight); LV/TL (left ventricle/tibia length); ANP (atrial natriuretic peptide); BNP (brain natriuretic peptide); RAS (renin angiotensin system); Col1 (collagen 1); CTGF (connective tissue growth factor); TMAO (trimethylamine N-oxide); GF (germ-free); FMT (fecal matter transplant)
